# Heterogeneity of expression of epithelial–mesenchymal transition markers in melanocytes and melanoma cell lines

**DOI:** 10.3389/fgene.2013.00097

**Published:** 2013-05-31

**Authors:** Ji Eun Kim, Euphemia Leung, Bruce C. Baguley, Graeme J. Finlay

**Affiliations:** Auckland Cancer Society Research Centre, Faculty of Medical and Health Sciences, The University of AucklandAuckland, New Zealand

**Keywords:** E-cadherin, Axl, MITF, melanocyte, melanoma

## Abstract

The epithelial–mesenchymal transition (EMT) describes a reversible switch from an epithelial-like to a mesenchymal-like phenotype. It is essential for the development of the normal epithelium and also contributes to the invasive properties of carcinomas. At the molecular level, the EMT transition is characterized by a series of coordinated changes including downregulation of the junctional protein E-cadherin (*CDH1*), up-regulation of transcriptional repressors of E-cadherin such as Snail (*SNAI1*) and Slug (*SNAI2*), and up-regulation of N-cadherin. We wished to determine whether cultured normal melanocytes and melanoma cell lines, which are derived from the neural crest, showed signs of a similarly coordinated phenotypic switch. We investigated normal melanocytes and 25 cell lines derived from New Zealand patients with metastatic melanoma. Most lines had been previously genotyped for common mutations such as *BRAF*, *NRAS*, PIK3CA (phosphatidylinositol-3-kinase), *TP53* (p53), and *CDKN2A* (p16). Expression of E-cadherin, N-cadherin, microphthalmia-associated transcription factor (MITF), Snail, Slug, Axl, p53, and Hdm2 was compared by western blotting. Normal melanocytes expressed each of these proteins except for Snail, while normal melanocytes and almost every melanoma line expressed Slug. Expression of individual markers among different melanoma lines varied from high to low or undetectable. Quantitation of western blots showed that expression of MITF-M, the melanocyte-specific isoform of MITF, was positively related to that of E-cadherin but inversely related to that of N-cadherin and Axl. There was also no apparent relationship between expression of any particular marker and the presence of *BRAF*, *NRAS*, *PIK3CA*, *TP53*, or *CDKN2A* mutations. The results suggest that melanomas do not show the classical epithelial and mesenchymal phenotypes but rather display either high E-cadherin/high MITF-M expression on one hand, or high N-cadherin/high Axl expression on the other. These may correspond to differentiated and invasive phenotypes *in vivo*.

## INTRODUCTION

The epithelial–mesenchymal transition (EMT) describes a reversible phenotypic change in epithelial cells that is essential for embryogenesis and wound healing in normal tissues. It is characterized by the loss of functional E-cadherin containing junctions and loss of cell polarity, and is particularly associated with the expression of zinc-finger transcription factors Snail (*SNAI1*) and Slug (*SNAI2*), as well as of ZEB1 (zinc-finger E-box-binding homeobox 1), ZEB2, FoxC2 (forkhead box protein C2), and TWIST (Lim and Thiery, 2012). Expression of the intermediate filament protein vimentin appears to be upregulated by Slug in cells undergoing EMT; vimentin then up-regulates the Axl tyrosine kinase, which contributes to changes in cytoskeletal architecture and migratory potential (Ivaska, 2011). These changes in adhesion proteins cause cells to change to a morphology resembling that of mesenchymal cells and to a functional change toward migration, invasion, and resistance to apoptosis. Evidence for EMT has also been found in carcinomas, leading to the proposal that it is involved in both invasion and metastasis (Lim and Thiery, 2012).

Melanocytes differ from epithelial cells in having their origin in the neural crest, a collection of multipotent and migratory cells in the vertebrate embryo that is also important for the development of cartilage, bone, neurons, glia, and smooth muscle. Although the term EMT arose from studies in epithelial tissues, it has been applied to a variety of developmental tissues including migratory neural crest cells that are the precursors of melanocytes. Slug appears to be essential for precursor migration and melanocyte development in mammals; Slug knockout mice exhibit some features of the Waardenburg syndrome in humans, which is associated with hypopigmentation and hearing loss (Shirley et al., 2012), while loss of one Slug allele in humans is associated with piebaldism (Sanchez-Martin et al., 2003). Expression of Slug is closely related to that of microphthalmia-associated transcription factor (MITF; Sanchez-Martin et al., 2002), which in turn is essential for expression of proteins mediating the production of melanin by mature melanocytes. Such cells also express E-cadherin, presumably allowing both functional interaction with E-cadherin expressed on keratinocytes (Kuphal and Bosserhoff, 2012) and transfer of melanosomes.

Melanoma cells differ from melanocytes by acquiring invasive and/or metastatic properties, depending on the state of the melanoma (Orgaz and Sanz-Moreno, 2013). It has been suggested that the invasive and metastatic potential of melanoma cells reflects their ability to undergo EMT-like reversible phenotypic changes (Shirley et al., 2012). Histological studies of melanoma show frequent expression of Slug, E-cadherin, and MITF but also considerable heterogeneity of expression of these proteins among individual cells from the same specimen (Shirley et al., 2012). The aim of this study was to assess the degree of coordinated expression of EMT-associated markers in a series of low passage human melanoma cell lines, comparing expression with that of cultured normal melanocytes. We utilized a series of melanoma lines that were originally derived from New Zealand patients with metastatic melanoma to assess responses to radiotherapy and chemotherapy (Marshall et al., 1992, 1994; Kim et al., 2012). Many of these cell lines have been characterized for genetic mutations in *BRAF*, *NRAS*, *PIK3CA* (phosphatidylinositol-3-kinase), *TP53* (p53), and *CDKN2A* (p16) genes (Parmar et al., 2000; Charters et al., 2011). In this study, we have grown 25 of these melanoma cell lines, characterized their expression of E-cadherin, N-cadherin, Snail, Slug, Axl, p53, Hdm2, and MITF, examining the relationship between protein expression and common genetic aberrations.

## MATERIALS AND METHODS

### CULTURE OF MELANOMA CELLS AND MELANOCYTES

The 25 New Zealand melanoma (NZM) cell lines were generated from surgical samples of metastatic melanoma as previously described (Marshall et al., 1994; Kim et al., 2012). Written consent was obtained from all patients under Auckland Area Health Board Ethics Committee guidelines. NZM cell lines were grown under low oxygen conditions (5% O_2_) in order to mimic physiologically low oxygen levels in tumors. NZM lines were grown in α-modified minimal essential medium (αMEM; Invitrogen, USA) supplemented with insulin (5 μg/mL), transferrin (5 μg/mL), and sodium selenite (5 ng/mL; Roche Applied Sciences, Germany), 100 U/mL of penicillin, 100 μg/mL of streptomycin (PS), and 5% fetal bovine serum (FBS). Human primary melanocytes were purchased from Invitrogen and grown in light sensitive Medium 254 supplemented with human melanocyte growth supplement (HMGS-2; Invitrogen) and PS. Human melanocytes were cultured in an atmosphere of 5% CO_2_ in air at 37°C. Genetic analyses of *BRAF*, *NRAS*, *TP53*, *CDKN2A*, and *PIK3CA* in NZM cell lines were carried out. Selected melanoma cell lines were sequenced for mutations in *BRAF*, *NRAS*, and *PIK3CA* as previously described (Kim et al., 2012). Sequencing for mutations in the *TP53* and *CDKN2A* genes has been previously described (Parmar et al., 2000; Charters et al., 2011).

### WESTERN BLOTTING

After NZM cells were grown to about 80% confluence, they were washed in ice-cold phosphate buffered saline (PBS), lysed in radioimmunoprecipitation assay buffer and prepared for western blotting as previously described (Kim et al., 2009). Antibodies used were specific for the following epitopes: E-cadherin, N-Cadherin, Snail, Slug, and Axl were from Cell Signaling Technology; MITF was from Abcam; and p53, HDM2, and β-actin were from Santa Cruz. Western blots were quantified using Image J software and expressed as a ratio to β-actin.

### STATISTICAL ANALYSIS

Spearman’s rank correlation coefficient (*r*_s_) and statistical significance (*p*) were calculated using standard methods (SPSS). Values of *p* < 0.05 were considered to be statistically significant. Correlation plots were also fitted with best-fit hyperbolae.

## RESULTS

### EXPRESSION OF E-CADHERIN, N-CADHERIN, Snail, and Slug

**FIGURE 1:**
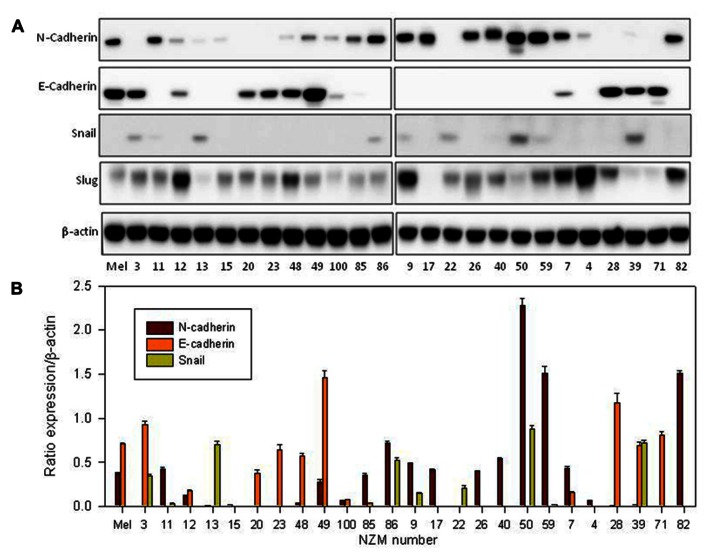
**(A)** Western blots of whole-cell extracts derived from cultures of normal melanocytes and of a number of melanoma lines, indicating expression of N-cadherin, E-cadherin, Snail, and Slug. The numbers indicate the identities of members of the New Zealand melanoma collection (e.g., 3 = NZM3); Mel indicates data for normal melanocytes. The western blot shown is representative of three independent repeats. **(B)** Western blot quantification of E-cadherin, N-cadherin, and Snail as ratios to β-actin loading controls. Bars show SEM.

**FIGURE 2:**
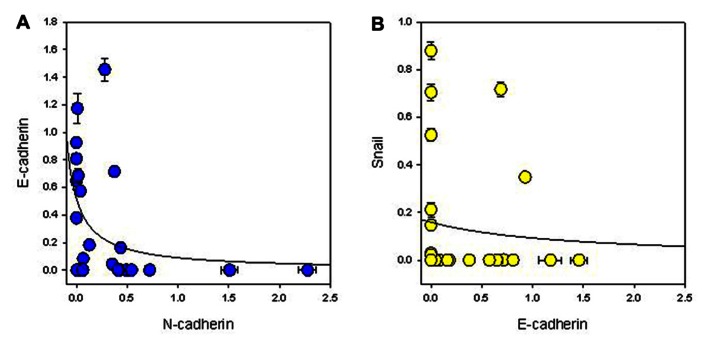
**Relationship between expression of (A) N-cadherin and E-cadherin, and (B) E-cadherin and Snail normalized to β-actin expression**. The lines indicate best-fit hyperbolae.

Since EMT is normally associated with loss of E-cadherin expression and gain of N-cadherin, we first measured cadherin expression. Normal melanocytes expressed both proteins and about half of the lines (NZM11, NZM85, NZM86, NZM9, NZM17, NZM26, NZM40, NZM50, NZM59, NZM4, and NZM82) showed moderate to strong N-cadherin expression but no E-cadherin expression. The other lines all expressed E-cadherin except for NZM22, which expressed neither (**Figure 1A**). When we quantified the western blots and normalized it to β-actin expression (**Figure 1B**), we observed an inverse correlation between E-cadherin and N-cadherin expression (**Figure 2A**). Quantification and statistical analysis showed a significant negative correlation between E-cadherin and N-cadherin expression (*r*_s_ = -0.578; *p* = 0.002). Slug, the putative transcriptional repressor for E-cadherin, was expressed in normal melanocytes as well as in all lines with the exception of NZM17. The relative expression of E-cadherin and Snail suggested an inverse correlation (**Figure 1A**). However, quantification (**Figure 2B**) showed this to be not statistically significant (*r*_s_ = –0.272; *p* = 0.18). We also tested whether expression of these markers was associated with any of the mutations shown in **Table 1**, but no clear relationship was found.

**Table 1:** Genetic status of the melanoma lines used in this study.

NZM	*BRAF*	*NRAS*	*TP53*	*CDKN2A*	*PIK3CA*
3	V600E			Deletion	
4	V600E		241S/P		
7	V600E		241S/P/WT		
9			179C/T	Deletion	
11	V600E			Deletion	
12	V600E				
13				Deletion	
15		Q61K			
17		Q61K	241S/T		
20	V600E			Deletion	
22			241S/T/W		
23					
26	V600E		136A/G		
28			241S/T/WT + 159a/v		
39			213A/G	Deletion	
40		Q61H	Del 249-253		H1047R
48		Q61K			
49	V600E			Deletion	
50			R280T		
59			Silent T/G	Deletion	
71					
82					
85					
86					
100					

### EXPRESSION OF Axl, MITF, p53, and Hdm2

**FIGURE 3:**
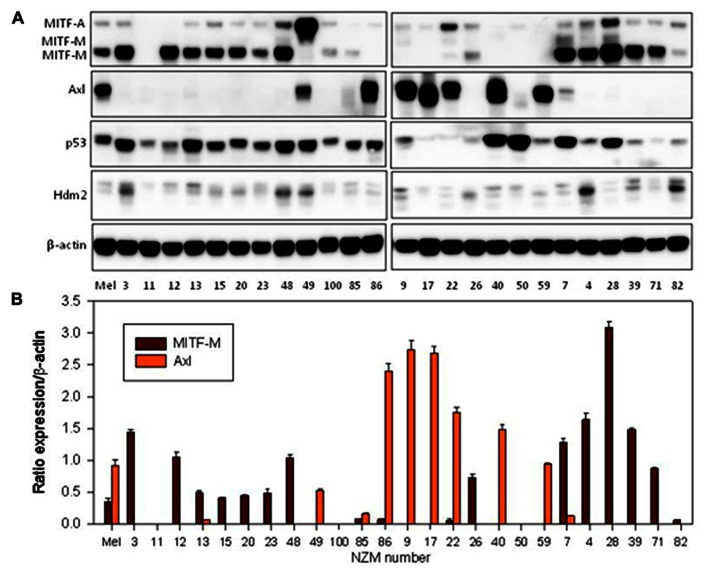
**(A)** Western blots of extracts from cultures of normal melanocytes and of a number melanoma lines, indicating expression of MITF-M (bottom two bands), MITF-A (top band), Axl, p53, and Hdm2. The numbers indicate the identities of members of the New Zealand melanoma collection; Mel indicates data for normal melanocytes. The western blot shown is representative of three independent repeats. **(B)** Western blot quantification of MITF-M and Axl as ratios to β-actin loading controls. Bars show SEM.

**FIGURE 4:**
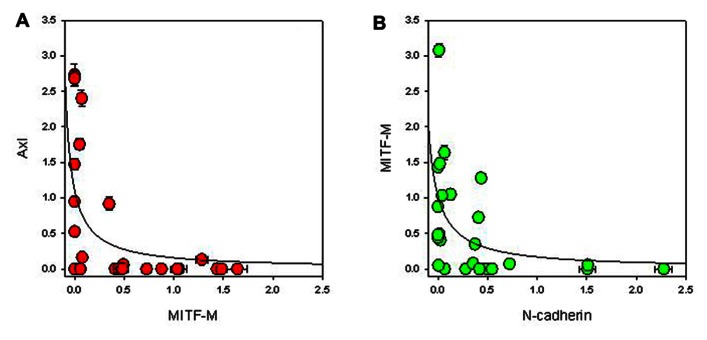
**Relationship between expression of (A) MITF-M and Axl, and (B) MITF-M and N-cadherin normalized to β-actin expression**. The lines indicate best-fit hyperbolae.

**FIGURE 5:**
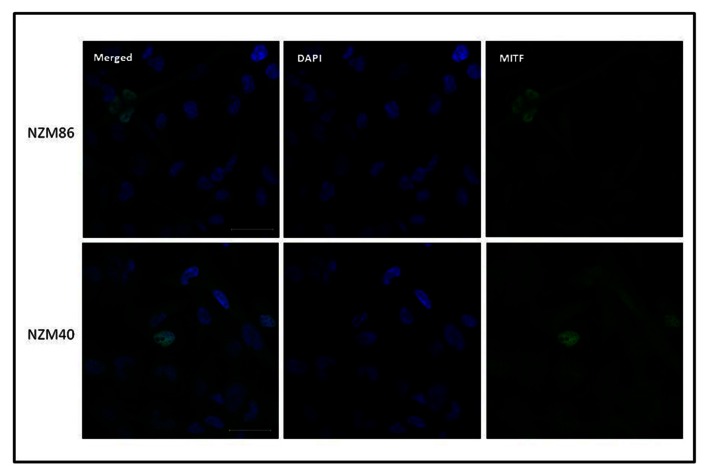
**Immunofluorescent images of NZM40 and NZM86 stained for MITF (green) and for DNA (diaminophenylindole: blue)**. Scale bars on the merged image indicate 50 μm.

It has been previously reported that EMT is associated with increased Axl expression (Gjerdrum et al., 2010) and reduced MITF expression (Sensi et al., 2011). We measured Axl expression and found it only in a proportion of cell lines (**Figure 3A**). Although it appeared from western blots that Axl expression was inversely correlated to E-cadherin expression, quantitation failed to show significance (*r*_s_ = -0.108). MITF has several isoforms (Yasumoto et al., 1998), and the A and M isoforms are expressed in the melanocyte lineage (Goding, 2000) with the M isoform having differentially spliced variants (Hodgkinson et al., 1993; Steingrimsson et al., 1994; Selzer et al., 2002). Both MITF-A and MITF-M were found in the cell lines (**Figure 3**), with the MITF-M isoform appearing as two differentially spliced variants. We quantified blots for MITF isoforms (**Figure 3B**) and observed a statistically significant inverse relationship (*p* = 0.006) between MITF-M expression and Axl expression (**Figure 4A**). Several cell lines (NZM49, NZM22, and NZM7), as well as melanocytes, expressed both Axl and MITF. Interestingly, NZM49 and NZM22, which express both MITF and Axl, expressed more MITF-A than other cell lines. Furthermore, there was a significant negative correlation between MITF-M and N-cadherin expression (*r*_s_ = –0.562; *p* = 0.007; **Figure 4B**) and a significant positive correlation between MITF-M expression and E-cadherin (*r*_s_ = 0.514; *p* = 0.007; not shown). Since it has been reported that loss of p53 expression is associated with EMT (Gadea et al., 2007), we also measured expression of p53 and of Hdm2, a protein closely associated with p53 degradation (Araki et al., 2010). However, there was no obvious relationship between expression of either p53 or Hdm2 and that of other EMT markers (**Figure 3A**). As MITF has been noted to be one of the key molecular switches that determine switching of different cell progeny (Cheli et al., 2011), we also stained for MITF to observe expression in individual cells within the same cell line. Interestingly, in NZM86 and NZM40 (two cell lines that express very low MITF as determined by western blotting) we observed individual cells that expressed detectable levels of MITF (**Figure 5**) scattered amongst low MITF expressing cells.

## DISCUSSION

The analysis of this series of early passage human melanoma lines has shown them to be highly heterogeneous not only with respect to expression of proteins directly associated with EMT such as E-cadherin, Snail, Slug, and Axl (**Figure 1**) but also with respect to expression of proteins that are more indirectly associated with EMT, such as MITF and p53 (**Figure 3**). Melanoma lines (with one exception) and normal melanocytes, expressed Slug. Other markers are generally strongly expressed in some lines but not others. Among the melanoma lines, we found that expression of MITF-M, the melanocyte-specific isoform of MITF, was positively related to that of E-cadherin but inversely related to that of N-cadherin and Axl (**Figures 4A,B**). A possible interpretation of the results is that melanoma lines show mesenchymal properties overall, but that individual lines vary between a high E-cadherin/high MITF-M expression and a high N-cadherin/high Axl expression phenotype. Cultured normal melanocytes show an intermediate phenotype, expressing all markers.

The results agree with an earlier study reporting that Axl-positive melanoma cells do not express MITF (Sensi et al., 2011). They also support a previous study that used a series of NZM melanoma cell lines to identify a gene expression signature that distinguished two phenotypes differing in their *in vitro* invasive potential (Jeffs et al., 2009). Although the cell lines used in that study overlap only partially with the lines used in the present study it is evident that the six lines with the “non-invasive” signature (NZM3, NZM4, NZM7, NZM12, NZM15, and NZM20) expressed MITF but little or no Axl while four with the “invasive” signature (NZM9, NZM11, NZM22, and NZM40) expressed no MITF but often expressed Axl (**Figure 3**).

One of the important questions posed in this study is whether the pattern of expression of proteins in the EMT pathway is related to genetic mutation. A detailed analysis of the mutational status of the melanoma lines will be reported elsewhere in this issue (Stones et al., 2013) but with the available data shown in **Table 1**, we have been unable to detect any significant relationship between expression of proteins shown in **Figures 1** and **3** and the mutational status of *BRAF*, *NRAS*, *TP53*, *CDKN2A*, or *PIK3CA*. These results echo those obtained from a study on the utilization of enzymes in the PI3K–PKB (phosphoinositide 3-kinase–protein kinase B), MEK–ERK (mitogen-activated protein kinase kinase–extracellular signal-regulated kinase), and mTOR–p70S6K (mammalian target of rapamycin–p70 ribosomal S6 kinase) signaling pathways. As determined by phosphorylation of signaling components, phosphorylation varied widely across a series of cell lines but did not directly reflect the *PIK3CA*, *PTEN*, *NRAS*, or *BRAF* mutational status of genes of these lines (Kim et al., 2012). A feature of the results is that individual melanoma lines vary enormously in their expression of particular proteins. This extends a previous study showing a large amount of heterogeneity in expression of MITF and the melanocyte lineage proteins PAX3 across a series of NZM lines, with cellular protein levels varying by 15-fold and more than 100-fold, respectively (He et al., 2011). Phenotypic switching has previously been suggested to explain differences in transcription signatures that correspond to different cellular phenotypes (Hoek et al., 2008; Hoek and Goding, 2010) and could account for the differences in protein expression.

Recently, MITF has been suggested to be crucial in determining whether melanoma cells proliferate (melanoma initiating cells) or change to accommodate a more invasive phenotype (Carreira et al., 2006; Hoek and Goding, 2010; Cheli et al., 2011); this has formed the basis for the hypothesis discussed separately in this issue (Eccles et al., unpublished). The mechanistic basis of such switching has not yet been elucidated but the concept is consistent with evidence that melanomas cells do not have a defined hierarchical organization with stem cells at one end and differentiated cells at the other (Quintana et al., 2008). Rather, each cell in a population may have a certain probability of switching to or from a phenotype with stem cell characteristics. There are speculations as to what could induce or decrease MITF activity (Strub et al., 2011) and determine the invasiveness or the stemness of the melanoma cells in response to hypoxia (Cheli et al., 2012) or to other factors in the tumor microenvironment (Li et al., 2003). One interesting observation is that even though NZM40 and NZM86 show low MITF expression by western blotting, we clearly see by microscopy that some cells highly express MITF (**Figure 5**), which is evidence of a heterogeneous population of cells (Quintana et al., 2010).

**FIGURE 6:**
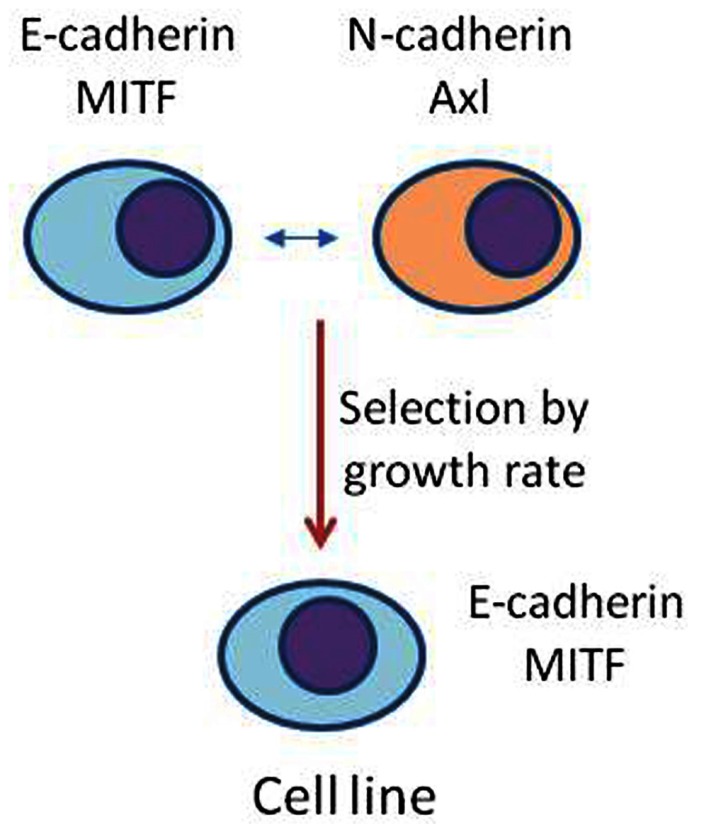
**Possible model for the generation of melanoma cell lines**. Phenotypic switching *in vivo* generates a highly heterogeneous population of cells that vary in expression of proteins such as E-cadherin, N-cadherin, Axl, and M-MITF. Derivation of a cell line, by selecting for rapid proliferation, may select for an individual phenotype.

Histological studies on *in vivo* human melanoma tissue have shown considerable heterogeneity by individual cells in expression of markers associated with EMT (Shirley et al., 2012) and this is consistent with the *in vitro* histological data shown in **Figure 5**. It is possible that melanoma tissue *in vivo* shows even greater phenotypic diversity than the derived cell lines. Thus, as shown diagrammatically in **Figure 6**, the *in vivo*, population develops, by phenotypic switching, a diverse population with individual cells exhibiting a high E-cadherin/high MITF-M expression on one hand or a high N-cadherin/high Axl expression on the other. Melanomas *in vivo* generally have cell cycle times of approximately 1 week, while derived cell lines have cell cycle times of 1–2 days (Baguley, 2011). Development of cell lines thus exerts a strong selective pressure for outgrowth of more rapidly cycling cells and may tend to select one of these phenotypes. Thus, melanoma tissue may be characterized as a mixture of phenotypes, some expressing high MITF-M and E-cadherin with more differentiated non-invasive behavior, and others expressing high N-cadherin, Slug, and Axl and with a more invasive behavior.

## Conflict of Interest Statement

The authors declare that the research was conducted in the absence of any commercial or financial relationships that could be construed as a potential conflict of interest.
